# The Implications of Meno’s Paradox for the Mental Capacity Act 2005

**DOI:** 10.1093/medlaw/fww026

**Published:** 2016-12-22

**Authors:** Paul Skowron

**Affiliations:** Centre for Social Ethics and Policy, University of Manchester, Manchester, UK

**Keywords:** Advocacy, Care Act, Epistemology, Mental Capacity, Paradox, Decision-Making

## Abstract

Meno’s paradox—which asks ‘how will you know it is the thing you didn’t know?’—appears in Plato’s dialogue of the same name. This article suggests that a similar question arises in some supportive relationships. Attention to this question clarifies one condition necessary to justify making a best interests decisions against someone’s will: the decided-for person must be unable to recognise that they have failed to recognise a need. From this condition, two duties are derived: a duty to ensure that someone cannot recognise that they have failed to recognise a need before making a decision against their will; and a duty to provide consensual support to those who have had decisions made against their will, in order to help them to avoid such second-order failures of recognition in the future. The article assesses the Mental Capacity Act 2005 against each of these duties. For each duty, it finds that the Act allows compliance, but does not robustly require it.

## 1. Introduction

When, if ever, is it right to make a decision for another person that they object to? The question seems to be intractable. The Mental Capacity Act 2005 (the MCA) provides one set of answers, but the UN Convention on the Rights of Persons with Disabilities (the UNCRPD)^1^ offers another, apparently incompatible, set.^2^ The MCA says that such a decision can be made if it is in someone’s best interests and they cannot understand, retain, or use and weigh the relevant information due to ‘an impairment of, or a disturbance in the functioning of, the mind or brain’.^3^ The UNCRPD, in contrast, has been interpreted to prohibit all ‘substituted decision-making’: defined as any process in which a third party is appointed against someone’s will to make decisions for them; and the decision is made in the person’s best interests, rather than according to the best interpretation of their will and preferences.^4^ Compelling points have been made against both of these positions, and each has been given increasingly subtle interpretations to accommodate these objections.^5^ The resulting dialogue, however, does little to dispel the impression that the core question is intractable; for, on this, neither side has appreciably changed its answer.

This article does not solve the question of whether it is better to make decisions for others in their bests interests or according to the best interpretation of their will and preferences. Instead, it draws attention to a paradox that can occur in supportive relationships. From this paradox, this article shows that best interests decision-making against a person’s will, *if* it is justified in a liberal society, must be premised on the decided-for person being unable to recognise that they have failed to recognise a need. From this premise, two duties can be derived; and this allows any particular system that uses a best interests standard, in this case the MCA, to be evaluated against standards that it can be assumed to be implicitly committed to. This is not the same task as assessing the Act against an external normative framework, such as the one that the UNCRPD presents. Nevertheless, it is striking that if these duties were taken seriously, then it would narrow, without entirely closing, the controversial gap between the two systems.

The next section develops an account of the paradox. Section III derives the first duty, the duty of identification, and assesses the MCA against it. Section IV then evaluates the extent to which legal requirements to provide advocacy might require compliance with this duty. Finally, Section V derives the second duty, the duty to support, and assesses the Act against this. For each duty, it is found that the MCA allows compliance, but stops some way short of requiring it. In the conclusion, these limited criticisms of the Act are reintegrated into the context of the wider debate.

## II. MENO’S PARADOX IN SUPPORTIVE RELATIONSHIPS

Meno’s paradox is presented by Plato in the dialogue of the same name. It is stated in two ways: first by Meno and then by Socrates.^6^ Socrates’ statement of the problem is slightly clearer. He says:

[A] man cannot search either for what he knows or what he does not know… He cannot search for what he knows – since he knows it, there is no need to search – nor for what he does not know, for he does not know what to look for.^7^

A common response to this paradox is to deny that knowledge is as binary as it suggests. So, for instance, Fine interprets Plato’s Socrates as proposing a distinction between ‘knowledge’ and ‘true beliefs’.^8^ On this interpretation, we may believe things to be true that are actually true without knowing why they are true.^9^ These true beliefs then provide us with a starting point from which we can develop a deeper understanding;^10^ and this understanding may, in time, develop to the point where we can give a coherent account of why our beliefs are true. It is this ability to explain ‘why’ that distinguishes knowledge from belief.^11^ There is, however, a form of the paradox that this distinction does not resolve.

Meno originally asks Socrates ‘how will you know it is the thing you didn’t know?’^12^ This suggests a particularly difficult problem, the problem of recognition.^13^ If knowledge requires being able to give an account of the reasons why a belief is true, then those ‘reasons’ will inevitably refer to other beliefs that, to count as knowledge, must in turn rely on even deeper reasons. There is no obvious end to this process, and so no certain way of distinguishing between what you know and what you falsely believe that you know.^14^ There is always the possibility that something we take to be knowledge is actually premised on a deep unrecognised error. Sometimes, this is directly relevant to supportive relationships.

A paradox of recognition can occur in supportive relationships. This can be shown by an example. Imagine a man called ‘John’, who has a large (over 9 cm) abdominal aortic aneurysm that is growing rapidly. John wants to live. The diagnosis of such an aneurysm is reliable, they are very likely to rupture, and rupture carries an extremely high risk of death.^15^ Assuming, for now, that there are no other complicating factors, it is reasonable to say that John objectively needs a particular form of support: surgery. Further imagine, however, that John does not recognise his need for this support. He is asymptomatic, and simply disbelieves the diagnosis. In these circumstances, those supporting him might want to help him to recognise that if he wishes to live, he needs surgery. This is when the paradox appears. If John accepts this help—a second-order support to recognise his need for first-order support—then that may give us a reason to suspect that he is not as certain as he seemed about not needing surgery. To accept the second-order support shows some recognition of the fact that he may have the first-order need, surgery. This is the paradox; and, more importantly, it also works in the opposite direction. If John truly does not recognise his need for surgery, then he will not recognise that he has a reason to accept help to recognise that need. If a supporter says, ‘we want to help you to see that you need surgery’, then he is likely to reply ‘but I don’t need surgery’. Unless he is a particularly patient man, this might be followed by a comment about how nobody is listening to him.

To this use of the paradox, it might be objected that John could recognise his need for surgery yet refuse it. This raises two possibilities. An example of the first would be if John wished to live, and understood that he needed surgery to do so, but nevertheless decided that a religious prohibition made it impossible for him to accept the intervention. In this case, however, there is good reason to doubt whether John truly needs the surgery, no matter what his medical needs might be.^16^ In contrast, an example of the second possibility would be if John wanted to live, and accepted that he needed surgery to live, but nevertheless refused the intervention without being able to give any reason why. In this case, however, what has occurred is not recognition. The concern in these situations is with a practical question, not with someone’s ability to assent to theoretical propositions; so ‘recognition’ here necessarily includes the ability to make practical use of what has been recognised. This roughly accords with the natural use of the language. When, in this example, John agrees that he needs surgery to live, he has clearly assented to a proposition. After he refuses the surgery for no discernible reason, however, it would be unusual to nevertheless say that he ‘recognises’ his need for surgery. In conclusion, in the first example, John recognises an apparent need but denies that in the circumstances it is a need for him; and in the second what appears to be recognition is better characterised as mere assent. In neither case has John recognised a need for him and yet refused surgery.

Examining this paradox can help to reveal one condition necessary to justify systems, such as the MCA, that allow decisions to be made in someone’s best interests against their will. For such decisions to be justified, it is not enough that someone fail to recognise a need; they must also fail to recognise that they have failed to recognise the need. If a person can recognise their own first-order needs, then they are capable of recognising their own best interests; so, in a liberal society, there is no obvious justification for giving someone else the power to make such decisions for them. Beyond this, however, if someone does not recognise a first-order need but knows that they do not do so, then there is still no clear justification for giving someone else the ability to make decisions for them *against their will*. If, for example, John says ‘I must be missing something, you decide for me’, then the decision is not against his will. If, in contrast, he says ‘I must be missing something, please explain it to me again’, then there is no clear justification for ignoring his request for support and simply making the decision. When someone recognises their second-order need for help to identify their first-order needs, then less intrusive measures than a decision against their will are necessarily available. In other words, if it is assumed that self-determination is generally good for people and that any legal suspension of it should be for a reason, then the mere fact that people sometimes have needs that they cannot recognise is not enough to justify best interests decisions against someone’s will. If such decisions are justified, it must be because sometimes people fail to make the second-order recognition that they cannot recognise a first-order need. That is to say, systems that allow best interests decisions against a person’s will are only justified if people are sometimes caught in the paradox.

The argument in the previous paragraph does not demonstrate that best interests decisions against a person’s will are ultimately justified. It may be that even if someone is caught in the paradox, then making a decision against their will is still unjustified for other reasons. For example, it might always cause significantly more harm to the person than respecting their preferences would. For this reason, reflecting on the paradox cannot settle the clash between the MCA and the UNCRPD discussed in the introduction. Nevertheless, if a second-order failure of recognition is necessary for a best interests decision against someone’s will to be justified in a liberal society, then there are implications. In particular, the power to make such decisions will plausibly carry with it duties.

## III. THE DUTY OF IDENTIFICATION

The previous section suggests that if best interests decisions made against someone’s will are to be justified in a liberal society, then it is not enough that the person does not recognise a first-order need; they must also fail to recognise that they do not recognise a first-order need. From this general form of the argument, a specific form can be derived: in any particular case, a best interests decision against a person’s will is only justified if they both fail to recognise a first-order need and fail to recognise that they are doing so. If it is assumed that decisions should only be made when it is justified to do so, then from this specific form of the argument a duty can be derived: the duty to identify whether someone is making such a second-order failure of recognition before deciding against their will.

There is, however, a problem; for this duty can become ensnared in the paradox that it is derived from. If the duty is fulfilled, a decision is only made when the person cannot be helped to make the second-order recognition that they have failed to recognise a first-order need. If, though, the person, such as John in the original example, is in this position, then they will deny that this is an accurate description of their situation. In other words, the same conditions that are necessary to justify the power to make a decision also make the use of that power unlikely to be accepted. This has profound consequences. So far, this article has largely taken a need as a simple objective fact, but, of course, no supporter will ever have unmediated access to the objective facts. They will have, at best, a tangle of evidence from various sources about what someone’s needs are. Furthermore, even a person’s objective needs will be influenced by subjective factors. Even in the example, it was necessary to say that John wanted to live. A person’s experiences and values partially shape their needs. This creates an evidential problem, for each of us has an access to these subjective elements that an assessor cannot equal. Atkins puts the point well: ‘appreciation of the subjective character of experience brings with it the necessity for an epistemological humility’ on the part of others.^17^ If the supported person has better access to some of the evidence, then there is always the possibility that what appears to be a case of them failing to recognise a need is actually a case of third parties attributing needs to them that they do not have.^18^

Subjective elements shape our needs, so it will often be difficult to show that someone has needs that they are failing to recognise. This does not, however, mean that providing sufficient evidence is always impossible. Unfortunately, the UN Committee on the Rights of Persons with Disabilities appears to have concluded otherwise. It says that it is a flaw to presume to be able to ‘accurately assess the inner-workings of the human mind’.^19^ If this is a flaw it is, as Dawson points out, one that can be found throughout the legal system;^20^ but, beyond that, the Committee fails to apply its own position consistently. Some evidence is only accessible to the supported person, but this is seldom the only relevant evidence that is only accessible to one person. Imagine John says that he doesn’t need surgery, and supports his claim by saying that the doctors have kidnapped him in order to harvest his organs. In this case the staff, by virtue of *their* subjective experience, will have access to evidence that is not available to John. Some of this evidence, for instance, the doctor’s intention to save John’s life or her memories of learning how to interpret the relevant scans, will bear on the question of what John’s needs actually are. The existence of subjective perspectives does not, by itself, call into question the existence of objective needs. It just means that these situations are evidentially complex.

Evidentially complex situations are not unusual. As Wimsatt points out, ‘for the complex systems encountered in evolutionary biology and the social sciences, it is often unclear what is fundamental or trustworthy’.^21^ A common response is to seek robust results: if different types of measurement all return the same result, then that result can be treated as more trustworthy.^22^ This idea of seeking robust results is relevant here; for if the person concerned has access to evidence that cannot be gained in any other way, then this implies that their own assessment of the situation should never simply be ignored. This in turn suggests that the duty of identification is only fulfilled if the person’s own perspective on their needs is sought out and treated as direct evidence of whatever it is that they assert. Only if the countervailing evidence is overwhelming should the person’s view be treated as evidence that they cannot recognise an objectively existing need. In practice, this may equate to Banner and Szmukler’s application of Davidson’s Principle of Charity: ‘we must assume that a speaker is by and large consistent and correct in his beliefs’.^23^ Indeed, if a supporter takes the opposite approach, and *begins* by taking a person’s disagreement as evidence that they cannot identify their needs, then it is not the person who does not recognise that they have failed to recognise something. It is the supporter.^24^ The approach suggested here will not, of course, resolve every possible evidential problem. It cannot address the question of what should first trigger the suspicion that someone does not recognise their own needs. It can only suggest what should be done when that suspicion is already present. Similarly, it cannot help if it is suspected that someone cannot recognise a need, but, despite every available support, they cannot or will not share their own perspective. This last point can, however, be accommodated by acknowledging that the duty of identification requires starting with the person’s own perspective only when it is possible to do so.

If systems which allow best interests decisions against a person’s will should respect the duty of identification, and the duty of identification requires someone’s testimony to be treated as evidence of what they say before it is treated as evidence that they do not understand, then this offers two criteria by which the MCA can be assessed. The first criterion is whether it recognises the duty of identification at all, and the second is whether it recognises that the duty entails this careful treatment of the person’s own testimony. It meets the first criterion. Capacity assessments under the MCA are of someone’s ability to make a particular decision;^25^ and an inability to make a decision is specified as an inability to understand, retain, use, or weigh relevant information; or an inability to communicate the decision.^26^ The statutory language is wider than that derived from the paradox, but ‘relevant information’ includes the consequences of the decision,^27^ and this seems close to being unable to identify your needs in that particular situation. This impression is reinforced when the Act’s principles are examined. Section 1(2) requires that someone is assumed to have capacity unless the contrary is established. If being unable to understand or use information is analogous with the content of the duty of identification, then it is this section of the Act that makes it a duty to undertake a process of identification in every case. Furthermore, this principle is reinforced elsewhere in the Act. Section 5(1) requires that, for acts ‘in connection with’ care or treatment, ‘reasonable steps’ are taken to establish whether the person has capacity; and section 1(3) requires ‘all practicable steps’ be taken to help someone to make a decision before they are treated as unable to do so. This last section should distinguish between those who can be helped to recognise their needs and those who cannot, and only allow decisions in the latter case. The Act, especially this provision, has ‘not been widely implemented’;^28^ but it certainly recognises the existence of something broadly similar to the duty of identification. Unfortunately, the same cannot be said of the second criterion, the careful treatment of the person’s own testimony.

The Act’s near silence on the question of evidence prevents it from requiring that the person’s own testimony be treated with the care that the duty of identification demands. It does contain a few evidential prohibitions: incapacity cannot be established merely because of an unwise decision; or because of a person’s condition, behaviour, age, or appearance.^29^ It does not, however, indicate how the person’s own perspective should be treated. The Code of Practice examines capacity assessments in more detail than the Act,^30^ but it does not mention the person’s perspective in this context. Although it discusses the possibility of challenging a capacity assessment,^31^ the standard against which the assessment will be measured is that of the Act and Code;^32^ so this takes things no further. Worse yet, an ambiguity in the language of the Code may mislead. It says that ‘nobody can be forced to undergo an assessment of capacity’.^33^ At face value, this would appear to mean that people have the ability to veto their own capacity assessment; although framed in an unhelpfully confrontational way, this would mean that their perspective would have to be taken into account. The Code has not been read in this way, as the ‘*DD*’ series of cases illustrate. In these cases, there was consensus that ‘threats or attempts to force *DD* to agree to an assessment are not acceptable’,^34^ but it was nevertheless declared lawful to remove her from her home ‘by force’ to a place where assessment could take place.^35^ Her lack of cooperation with the subsequent assessment was respected;^36^ but, importantly, this did not prevent her from being found to lack capacity to make decisions about litigation, contraception, and sterilisation.^37^ In other words, *DD* could decline to take part in her capacity assessment, but that assessment took place without her anyway. There is no right to veto your own capacity assessment, and the Act contains no less extreme way to ensure that the person’s own testimony is given a fair hearing, so the MCA fails to require that the second criterion of the duty of identification is fulfilled.

It seems likely that the Act’s failure to ensure that someone’s own testimony is treated as evidence contributes to such testimony being discounted in practice. Empirical studies show that a disagreement between an assessor and the person assessed can sometimes be taken as evidence that the person ‘lacks insight’, without first taking the disagreement as a reason to question the validity of the assessment.^38^ A lack of insight appears to be strongly correlated with a finding of incapacity,^39^ so the uncritical use of this concept is likely to have consequences. Professionals using ‘insight’ uncritically have been corrected by the Court of Protection,^40^ but judges, too, sometimes seem to use the concept in an indiscriminate way.^41^ When this happens, a person’s disagreement can be discounted if they lack insight, and their disagreement is evidence that they lack insight;^42^ so it becomes ‘impossible to disbelieve a doctor and retain capacity’.^43^ Here, the duty of identification has become entangled in the paradox that it was derived from; and it is the assessor, not the person assessed, who has failed to recognise what it is that they do not recognise. The Act allows this situation to be avoided. It does not, however, require that it be avoided.

## IV. ADVOCACY AND THE DUTY OF IDENTIFICATION

Since the MCA was passed, the perspective of the decided-for person has become a more prominent issue; and the effects of this new prominence can already be seen in domestic law, particularly in changes to the legal framework governing the provision of advocacy. These developments bring the law closer to requiring compliance with the second criterion of the duty of identification, but there are, however, limits to what advocacy can achieve in this respect. This section discusses the recent legislation, then its limits. First, however, it examines the advocacy provisions of the MCA.

The MCA requires an Independent Mental Capacity Advocate (IMCA) to be appointed when someone is thought to lack capacity, and has no-one else to speak on their behalf; and faces serious medical treatment, a long-term change of accommodation, or a deprivation of liberty.^44^ IMCAs can challenge capacity decisions,^45^ so they offer an avenue for challenging assessments that ignore the person’s own testimony. Nevertheless, unless appointed under section 39D of the Act when someone is deprived of their liberty,^46^ they are consulted to help determine what would be in the person’s best interests.^47^ This leads to an ambiguity. If the IMCA believes that the person lacks capacity, then they might not challenge an assessment that the person disagrees with; for they might not believe that it is in the person’s best interests to do so.^48^ In other words, IMCAs are merely allowed, not required, to argue for the person’s own perspective.

The advocacy provisions in the Care Act 2014 are more robust than those in the MCA, and provide a useful contrast, as the trigger for advocacy is still framed in terms of ability to make a decision. The newer Act requires an advocate to be appointed during needs assessments, care planning, or safeguarding processes;^49^ if ‘the individual would experience substantial difficulty’ making a decision, and no-one else can represent and support them.^50^ This is a lower bar than in the MCA, for ‘substantial difficulty’ does not require the person to be thought to lack capacity;^51^ but a more relevant difference between the two Acts is in what, exactly, the advocate is required to do once appointed. Under the Care Act, an advocate is *required* to communicate the ‘views, wishes, or feelings’ of a person thought to lack capacity,^52^ and the statutory guidance reinforces this as a duty to put forward the person’s own case.^53^ This goes further than the MCA towards robustly fulfilling the second criterion of the duty of identification, and it may also influence the older Act. The Law Commission has suggested that IMCAs ‘be replaced by a system of Care Act advocacy’; this would incorporate the more robust standard into the MCA itself.^54^

Strengthening the law governing the provision of advocacy is a step towards requiring compliance with the duty of identification, but it has limits. These limits are of three types: limits of scope, limits in implementation, and cultural limits. The legislation itself has limited scope. Only certain decisions trigger the duty to appoint an advocate. Between the two Acts, many major decisions will be covered, but it is not clear that all will be; and, as has been acknowledged in Parliament, ‘minor’ decisions can be as important to the person concerned as ‘major’ ones.^55^ In practice, however, limitations in implementation are likely to be more severe than those of scope. Failures of implementation were the most consistent theme of the recent House of Lords Select Committee Report into the MCA,^56^ and IMCAs are not always appointed even when it is legally required.^57^ Legislation requiring advocacy of a certain level is not, by itself, sufficient to ensure that practice will consistently reach that level; and the current political and economic climate is relevant here. Local authority budgets have fallen by almost 40% in real terms over the last five years, and authorities have lost their ability to insulate social care from the effects of this drop in funding; yet the overall need for care is increasing dramatically.^58^ In these circumstances, advocacy may be neglected; and the provision of more ‘basic’ physical care prioritised.

Scope and implementation offer relatively contingent limits to advocacy. Changes to the legislation, or to practice and funding, could overcome them. There are, however, also cultural limits to the ability of an advocate to fulfil the duty of identification, and these will be harder to overcome. These cultural limits gain their force from the paradox, but to show this requires careful disambiguation. The example of John can help. If an advocate wants to represent John’s own perspective as faithfully as possible, then she faces a choice. John both wishes to live and wishes to refuse life-saving surgery. If she presents either of these wishes alone, then she will not fully represent John’s relevant views, but if she presents both, then she will undermine John’s case by emphasising its contradictions. In these cases, then, presenting what someone wants without explaining why they want it is insufficient. Instead, an advocate should present the person’s overall view, for example, John’s view that refusing surgery will not risk his life, and the *reasons why* that view should prevail.^59^

If an advocate attempts to present the reasons why someone’s view should prevail, then another choice reveals itself. This is the choice between only presenting John’s reasons for his view and also presenting other reasons for reaching the same view. With regards to the duty of identification, these are different things. If the advocate presents John’s reasons for his view, then she is helping to ensure that his perspective is treated as evidence, as the duty requires. If, however, she offers reasons which are not John’s for why John’s wishes should prevail, then a shift in argumentation has occurred. Proposing reasons that are not John’s does not help to show that John understands the relevant information and hence has capacity. Instead, the advocate is effectively arguing that regardless of whether John has capacity or not, his wishes should prevail. This is no longer about the duty of identification; it does not bear on the question of whether John recognises his needs. There are good reasons for keeping these two lines of argumentation distinct. Being found to have capacity is not the same as being found to lack capacity but nevertheless having your wishes followed. A finding of incapacity can carry costs to the person beyond simply not getting what they want: for instance, self-stigmatisation and loss of confidence.^60^ Beyond this, it is only by disambiguating between the two that the cultural limits on advocacy can be made clear.

The distinction between advancing someone’s own reasons for their view and presenting other reasons for why that view should prevail makes it easier to see the cultural limits on the ability of an advocate to fulfil the duty of identification. An advocate’s position will often, regrettably, give her more credibility than the person themselves, but this credibility has limits, and some of these limits are cultural. If, again, John is paranoid and believes that he has been kidnapped so that his organs can be harvested, but is actually ill in hospital, then an advocate arguing that his reasons for refusing treatment are literally true will not help him. She will only harm her own credibility. For a reason to be convincing, it must have some coherence with the account of the world that listeners assume to be true. This is not, by itself, a problematic limit: in John’s case, even a charitable treatment of his testimony should almost certainly conclude that he is failing to recognise a need. It does, however, indicate where the problematic cultural limits to advocacy are. Experimental psychology has shown the existence of implicit social biases. These biases are entirely distinct to someone’s explicit attitudes, they are robust, and they predict behaviour.^61^ In other words, people consistently act on biases that they deny, and may not even perceive. In particular, strong implicit biases against those with disabilities have been shown to be widespread, even among professionals who work closely with them.^62^ These biases, combined with the need for a person’s reasons to have some minimal coherence with commonly held assumptions to be credible, are sufficient to impose limits on advocacy’s power to fulfil the duty of identification. Recognising the existence of widespread unacknowledged bias entails recognising the risk that sometimes when a person’s reasons seem unconvincing, because they do not cohere with the commonly accepted view of the world, it is society, not the person, which has not recognised its failure to recognise significant facts. Furthermore, when the paradox reappears in this societal form, it limits advocates in two ways. First, advocates may be implicitly biased; for explicit opposition to discrimination does not guarantee that someone is not implicitly biased.^63^ Secondly, if the advocate is not biased, and others are, then arguing for the person’s perspective will risk the advocate’s credibility in the same way, if seldom to the same extent, that arguing John really was going to have his organs harvested did. The advocate will literally be arguing against what ‘everyone knows’. These cultural limits will be difficult to address: implicit biases may be difficult to change,^64^ and such biases against those with disabilities do not seem to have fallen in the last decade.^65^ They are, however, only limits. An area of freedom should remain in which an advocate can challenge biases without destroying their own credibility; and if that balance is difficult to find, then this is an argument for strong advocacy, not against it. Nevertheless, the cultural limits on advocacy, in combination with the limits of scope and in implementation, do mean that while stronger advocacy legislation is a step towards requiring that a person’s testimony is treated with due care, it is only one step. Others will almost certainly be needed.

## V. THE DUTY TO SUPPORT

In addition to the duty of identification, a duty to provide support can be derived from the paradox; and, as with the first duty, this offers a criterion by which legal systems that permit best interests decisions against a person’s will can be evaluated. At the outset, however, it should be stressed that ‘support’ in this context is limited to support of one particular, relatively narrow, type. It is support to someone who has had a decision made for them because of a second-order failure of recognition, which is directed at helping them to avoid such failures in the future. There are further limits on this support—in particular, it cannot be against the person’s will—that are discussed below. First, however, this section shows how the duty follows from the paradox.

As argued in Sections II and III, deciding against a person’s will can only be justified if someone cannot recognise that they do not recognise a need. At the same time, however, interfering with someone’s self-determination is understood in liberal societies to be wrongful. Indeed, this understanding motivates the general principles of both the MCA^66^ and the UNCRPD.^67^ On a simplistic reading, this general prohibition on interfering with someone’s self-determination is entirely displaced when their second-order failure of recognition justifies making a decision against their will. Such a reading is, however, *too* simplistic; for it collapses the difference between doing an unqualifiedly right act and doing a justified wrong.^68^ Making a decision against someone’s will because of their second-order failure of recognition is not an unqualifiedly right act. The very structure of this description denies it. It presents an apparent wrong, making the decision against their will; and then presents a reason, the failure of recognition, for nevertheless doing it. It is, at best, a justified wrong; and this is important. Justified wrongs are necessarily cases in which moral conflicts have occurred and been resolved; but, as Williams observes, such conflicts are not ‘all soluble without remainder’.^69^ One ‘remainder’ he examines is the regret that an ‘admirable moral agent’ might feel, even when they are acting as well as circumstances seem to allow.^70^ This is a good starting place; for it seems right to feel some regret if we have made a decision against someone’s will, even if we are sure that doing so is justified in the particular case. Furthermore, it seems right that, as Williams says,^71^ someone experiencing such regret might conclude that they ought to avoid such situations arising in the future. In the particular case, the moral conflict has arisen because someone has not recognised that they do not recognise a need; so a regretful decision-maker might conclude that, where possible, they should help the decided-for person to avoid such second-order failures of recognition in the future. This, then, is the derivation of the duty to support. If it is accepted that a decision against a person’s will is not an unqualifiedly good act but a justified wrong, that the appropriate response to inflicting this justified wrong is regret, and that the appropriate response to such regret is to attempt to avoid such situations occurring in the future, then there is an apparent duty once a decision has been made against someone’s will to attempt to help them to avoid second-order failures of recognition in the future. This is an endoxic derivation, it appeals to widely held evaluative beliefs;^72^ so it can be rejected by someone who denies regret is an appropriate response to inflicting a justified wrong, or denies that we should act on such regret. Nevertheless, neither of those positions seems particularly appealing, so it does have some force. The duty to support, however, also faces an internal challenge.

Just like the duty of identification, the duty to support can become ensnared by the paradox that it is derived from. If the justification for making a decision against someone’s will is that the person could not be helped to recognise their failure to recognise a need, then to claim that there is, nevertheless, a duty to provide this same help seems absurd, doomed to failure from the outset. This objection may hold true in some cases; and in those cases, if they can be reliably identified, there is no obvious duty to support. Few cases, however, are likely to be of this type. Just because we cannot help someone to recognise their own failure of recognition at one time does not mean that we cannot do so over a longer time period. Therefore, despite the entanglement of the paradox, the duty to support will still exist in most cases. It will, however, be limited in another way; for it cannot be used to justify decisions against a person’s will. Before discussing this limit, however, it is worth evaluating whether the MCA recognises the duty at all.

The MCA may seem to recognise the duty to support. After all, section 1(3) requires ‘all practicable steps’ be taken to support someone to make a decision; and section 4(4) that someone is encouraged and permitted to improve their ability to take part in decisions affecting them, insofar as that is ‘reasonably practicable’. These provisions do not, however, require fulfilment of the duty to support; and this is, in part, due to the same ‘decision-specific’ structure of the Act that buttresses the duty of identification. Section 1(3) only requires that someone be helped to make the particular decision in question. It does not create any duty that continues after the decision is made, but it is then that the duty of support arises. Similarly, section 4(4) requires that the person’s ‘ability to participate’ be supported during best interests decisions, but it, too, is specific to the particular decision being made. It does not create any on-going duty.^73^ When a cohesive team is working well with the person, then an on-going duty to support might, nevertheless, emerge from these sections. As Series points out, however, both decision-making and support under the MCA are ‘dispersed over a large number of disparate actors’,^74^ so the Act does nothing to encourage this sort of practice. Therefore, as with the duty of identification, the MCA allows the duty to support to be recognised, but does not require it.

It may be that the MCA is the wrong place to look for the duty to support. The provision of support, beyond that required for the immediate decision, could simply be beyond its remit. After all, as Lady Hale says, the Act is ‘concerned with enabling the court to do for the patient what he could do for himself if of full capacity, but it goes no further’.^75^ This point has been reinforced in the Court of Appeal: acting in someone’s best interests grants no additional power ‘to obtain resources or facilities from a third party’.^76^ Given this, it is worth again looking to the Care Act 2014. After all, it gives local authorities duties to promote ‘control by the individual over day-to-day life’^77^ and to ‘reduce the needs for care and support of adults in its area’.^78^ At first sight, it seems possible to read a duty to help someone to recognise their own needs into these provisions, but, unfortunately, the detail of the Act makes this unlikely to consistently happen. Local authorities only have a duty to provide for needs that meet the ‘eligibility criteria’,^79^ and these criteria refer only to first-order needs, such as maintaining nutrition.^80^ Some service providers may decide that supporting someone to identify their own needs in the long term is the best way to help them to meet those needs; and local authorities can provide services when they have no duty to do so.^81^ Nevertheless, as with the MCA, there is nothing in the Care Act that makes fulfilling the duty to support required, instead of merely allowed.

Beyond failing to require the duty to support, the MCA also fails to recognise the limits of support. Words like ‘support’ can be slippery; and it is easy to envisage a duty to help someone to recognise that they have not recognised their needs quietly becoming a power to force someone to accept our assessment of their needs. Berlin’s famous warning, ‘to manipulate men, to propel them towards goals which you – the social reformer – see, but they may not, is to deny their human essence’,^82^ is as apt here as it is anywhere. One way to avoid this slippage is to return to the paradox, and to remember that deciding against a person’s will is only justified when they do not recognise their failure to recognise a first-order need. If making a decision against someone’s will does not make it possible to satisfy the first-order need, then this justification fails. This is what happens with the duty to support. If we say ‘due to the paradox, John cannot be helped to recognise his need for surgery, so we must decide for him’, then a decision to operate can be implemented without any further input from John. In contrast, if we say ‘John cannot be helped to recognise his need to recognise his needs, so we must decide for him’, then we cannot make the second-order recognition happen without John’s input, no matter what we decide. The subjective character of recognition dictates that it can only be reached by minimally consensual means; so the duty to support cannot justifiably be extended to decisions against a person’s will. The MCA, however, does not distinguish between these two different situations; so it permits the meaning of ‘support’ to slip beyond reasonable bounds. For instance, in *Northamptonshire Healthcare NHS Foundation Trust* v *ML,*^83^ a young man, ‘ML’, was given an ‘opportunity to fulfil his potential’ to be independent. The ‘opportunity’ involved a detention of up to two years in hospital and involuntary treatment that he would undoubtedly find traumatic.^84^ It is unlikely that he experienced this as an opportunity or as support; and if he did not, then it must be doubted whether it really was these things. The MCA both fails to require respect for the duty of support, and fails to prevent ‘support’ sliding into unjustified coercion.

## VI. CONCLUSION

Meno’s paradox clarifies one condition necessary for the justification of a best interest decision against someone’s will. It suggests that it is not enough that someone has failed to recognise a first-order need, but they must also fail to recognise their own failure of recognition. From this necessary condition, two duties can be derived: a duty to identify that any particular person is in this position before deciding against their will; and a duty to support those decided for to avoid this situation in the future. Any particular legislation that allows best interests decisions against a person’s will can be measured against these duties. The MCA allows the fulfilment of these duties, but it does not robustly require them. Given this, it is perhaps unsurprising that the recent House of Lords report found the ‘empowering ethos’ of the Act’s general principles has yet to be realised.^85^ The failure to require compliance with the two duties is not due to those principles. It is, rather, due to the finer details of the Act and other legislation; some of which could, perhaps, be changed. The duty of identification requires that, during capacity assessments, the person’s testimony is treated primarily as evidence of what they assert and only secondarily as evidence that they cannot recognise their needs. Changes to the legal framework governing the provision of advocacy has already brought the law closer to doing this, but there are important limits to what advocacy can achieve by itself. The second duty, the duty to support, could be given a legal basis by making a person’s inability to recognise their other needs an eligible need under the Care Act. The MCA, however, also allows ‘support’ to slip into unjustified coercion, and this problem would have to be addressed separately.

This article shows that the MCA only allows, and does not robustly require, respect for two duties that any liberal system that allows best interests decisions against a person’s will should be committed to. It does not, however, demonstrate whether or not best interests decisions are ultimately justified; and, given this, it does not support either the maintenance of the status quo, or the abandonment of all best interests decision-making that has been called for under some interpretations on the UNCRPD. Nevertheless, Meno’s paradox also presents a challenge to those who favour the latter view; for they would appear to be committed to one of three following responses to it: either that no one is ever caught in the paradox; or that those who are so caught should be allowed to suffer whatever avoidable harms follow from their second-order failures of recognition; or that third parties can interpret someone’s ‘will and preferences’ in ways that contradict what the person actually says. A detailed examination of these positions is beyond the remit of this article, but the first response appears false, the second callous, and the third sinister. Despite this, taking seriously the response to the paradox discussed here would seem to narrow, without entirely closing, the gap between the MCA and the UNCRPD. It would require increased attention to the person’s perspective during capacity assessments, and at least one form of additional, consensual support. These are both things that align well with the overall aims and ethos of the UN Convention.

## Footnotes

1 Convention on the Rights of Persons with Disabilities (adopted 13 December 2006, entered into force 3 May 2008) 2515 UNTS 3.

2 Peter Bartlett, ‘The United Nations Convention on the Rights of Persons with Disabilities and Mental Health Law’ (2012) 75 Modern Law Review 752.

3 MCA 2005 s 1–4. An inability to communicate, under s 3(1)(d), is not relevant to a decision that someone objects to.

4 UN Committee on the Rights of Persons with Disabilities, ‘General Comment No 1’ (2014) UN Doc CRPD/C/GC/1 para 27.

5 Elionóir Flynn and Anna Arstein-Kerslake, ‘The Support Model of Legal Capacity: Fact, Fiction, or Fantasy?’ (2014) 32 Berkeley Journal of International Law 124; Wayne Martin and others, ‘Achieving CRPD Compliance’ (Essex Autonomy Project 2014) 10–13; John Dawson, ‘A Realistic Approach to Assessing Mental Health Laws’ Compliance with the UNCRPD’ (2015) 40 International Journal of Law and Psychiatry 70; Lucy Series, ‘Relationships, Autonomy and Legal Capacity: Mental Capacity and Support Paradigms’ (2015) 40 International Journal of Law and Psychiatry 80; Piers Gooding, ‘Navigating the “Flashing Amber Lights” of the Right to Legal Capacity in the UNCRPD: Responding to Major Concerns’ (2015) 15 Human Rights Law Review 45.

6 Plato, ‘Meno’ in John M Cooper (ed), *Plato: Complete Works* (GMA Grube tr, Hackett 1997) 80d80e.

7 ibid 80e.

8 Gail Fine, ‘Inquiry in the Meno’ in Richard Kraut (ed), *The Cambridge Companion to Plato* (CUP 1992) 200, 206.

9 Perhaps because we simply accept what an authority, such as a teacher, says. Alasdair MacIntyre, *Three Rival Versions of Moral Enquiry* (Duckworth 1990) 63.

10 Scott distinguishes between knowing ‘parrot fashion’ and ‘synoptically’. Dominic Scott, *Plato’s Meno* (CUP 2006) 102–03, 183–84.

11 Plato (n 6) 98.

12 ibid 80d.

13 Nicholas P White, *Plato on Knowledge and Reality* (Hackett 1976) 42–47.

14 Scott (n 10) 83–84.

15 FL Moll and others, ‘Management of Abdominal Aortic Aneurysms: Clinical Practice Guidelines of the European Society for Vascular Surgery’ (2011) 41 European Journal of Vascular and Endovascular Surgery S1.

16 This point is dealt with in detail in the next section, but for now it is enough to observe that someone’s objective needs cannot be identified without taking into account their own understanding of their life.

17 Kim Atkins, ‘Autonomy and the Subjective Character of Experience’ (2000) 17 Journal of Applied Philosophy 71.

18 Judges in the Court of Protection are aware of this danger. See, for example, *V v Associated Newspapers* [2016] EWCOP 21 [67]–[68] (Charles J).

19 General Comment (n 4) para 15.

20 Dawson (n 5) 74.

21 William C Wimsatt, *Re-engineering Philosophy for Limited Beings* (Harvard 2007) 56.

22 ibid ch 4. This process is not infallible. ‘Robust detection’ will break down if apparently independent measurements are not truly independent. Brett Calcott, ‘Wimsatt and the Robustness Family’ (2011) 26 Biology and Philosophy 284.

23 Donald Davidson, ‘Psychology as Philosophy’ in Donald Davidson (ed), *Essays on Actions and Events* (OUP 2001) 229, 238; cited in Natalie F Banner and George Szmukler, ‘“Radical Interpretation” and the Assessment of Decision-Making Capacity’ (2013) 30 Journal of Applied Philosophy 379.

24 Thanks to David Gibson for this point.

25 MCA (n 3) s 2(1); *PC* v *City of York Council* [2013] EWCA Civ 478, [2014] 2 WLR 1 [35] (McFarlane LJ).

26 MCA (n 3) s 3(1).

27 ibid s 3(4).

28 Select Committee on the Mental Capacity Act 2005, ‘Mental Capacity Act 2005: Post-Legislative Scrutiny’ (2013–14, HL Paper 139) para 79–83, 103–10.

29 MCA (n 3) s 1(4), 2(3). The word ‘merely’ is significant—these factors can have evidential force if they do not individually determine the question—*D* v *R (the Deputy of S)* [2010] EWCOP 2405, [2011] WTLR 449 [40] (Henderson J).

30 Department for Constitutional Affairs, *The Mental Capacity Act 2005 Code of Practice* (The Stationery Office 2007) ch 4.

31 ibid para 4.63–4.65.

32 ibid para 4.64.

33 ibid para 4.59.

34 *The Mental Health Trust v DD (No. 3)* [2014] EWCOP 13, 142 BMLR 156 [36] (Cobb J).

35 ibid [37]–[41].

36 *The Mental Health Trust v DD (No.4)* [2014] EWCOP 44 [5] and, [8]; The Mental Health Trust v DD (No. 5) [2015] EWCOP 4 [135] (Cobb J).

37 ibid *The Mental Health Trust v DD (No. 4)* [13]; *The Mental Health Trust v DD (No. 5)* [63], [80].

38 Val Williams and others, ‘Making Best Interests Decisions: People and Processes’ (Mental Health Foundation 2012) <http://www.mentalhealth.org.uk/content/assets/PDF/publications/best_interests_report_FINAL1.pdf?view=Standard> accessed 21 June 2015, 55–58; Charlotte Emmett, ‘Homeward Bound or Bound for a Home? Assessing the Capacity of Dementia Patients to Make Decisions about Hospital Discharge: Comparing Practice with Legal Standards’ (2013) 36 International Journal of Law and Psychiatry 73, 77.

39 GS Owen and others, ‘Mental capacity, diagnosis and insight in psychiatric in-patients: a cross-sectional study’ (2008) 39 Psychological Medicine 1389.

40 *KK* v *STCC* [2012] EWCOP 2136 [36],[64](Baker J).

41 *Islington LBC* v *QR* [2014] EWCOP 26, (2014) 17 CCL Rep 344 [96] (Batten DJ); *An English Local Authority* v *SW* [2014] EWCOP 43, [2015] COPLR 29 [20] (Moylan J).

42 Kate Diesfeld and Stefan Sjöström, ‘Interpretive Flexibility: Why Doesn’t Insight Incite Controversy in Mental Health Law?’ (2007) 25 Behavioural Sciences and the Law 85.

43 Neil Allen, ‘Is Capacity “In Sight”?’(2009) 19 Journal of Mental Health Law 165, 167.

44 MCA (n 3) ss 37–39E.

45 The Mental Capacity Act 2005 (IMCAs) (General) Regulations 2006 reg 7(1)(b).

46 *AJ v A Local Authority* [2015] EWCOP 5, (2015) 18 CCLR 158 [107–11].

47 MCA (n 3) ss 37(1)(b), 38(1), 39(1)(b), 39A(1)(b), 39C(1)(c).

48 Select Committee (n 28) para 167.

49 Care Act 2014 ss 67(1), (3), 68(1).

50 ibid ss 67(4) and 68(3).

51 Department of Health, ‘Care and Support Statutory Guidance’ (2014) paras 7.55, 7.58.

52 The Care and Support (Independent Advocacy Support) Regulations 2014 reg 5(7).

53 Statutory Guidance (n 51) para 7.43.

54 Law Commission, ‘Mental Capacity and Deprivation of Liberty: A Consultation Paper’ (Consultation Paper No 222, 2015) para 9.41.

55 Joint Committee on the Draft Mental Incapacity Bill, ‘First Report’(2002–2003, HL Paper 189-l, HC 1083-I) para 27.

56 Select Committee (n 28).

57 Department of Health, ‘The Seventh Year of the Independent Mental Capacity Advocacy (IMCA) Service’ (2015) <https://www.gov.uk/government/uploads/system/uploads/attachment_data/file/416341/imca-report.pdf> accessed 10 August 2015, 34.

58 National Audit Office, ‘Care Act First-Phase Reforms’ (2015–2016, HC 82) paras 1.6–1.9.

59 The same point is true of ‘wishes and feelings’ in best interests decisions: Laura Pritchard-Jones, ‘This Man with Dementia – Othering the Person with Dementia in the Court of Protection’, Medical Law Review (forthcoming).

60 Bruce J Winick, ‘The Side Effects of Incompetency Labelling and the Implications for Mental Health Law’ (1995) 1 Psychology, Public Policy, and Law 6.

61 Kristin A Lane and others, ‘Implicit Social Cognition and Law’ (2007) 3 Annual Review of Law and Social Science 427; Brian A Nosek and others, ‘Implicit Social Cognition: From Measures to Mechanisms’ (2011) 15 Trends in Cognitive Sciences 152.

62 Michelle Clare Wilson and Katrina Scior, ‘Attitudes Towards Individuals with Disabilities as Measured by the Implicit Association Test: A literature review’ (2014) 35 Research in Developmental Disabilities 294.

63 Lane and others (n 61) 431.

64 Jennifer A Joy-Gaba and Brian A Nosek, ‘The Surprisingly Limited Malleability of Implicit Racial Evaluations’ (2010) 41 Social Psychology 137.

65 Wilson and Scior (n 62) 319.

66 MCA (n 3) s 1.

67 UNCRPD (n 1) art 3(a).

68 Rosalind Hursthouse, *On Virtue Ethics* (OUP 2001) 44–49 discusses this distinction.

69 Bernard Williams, ‘Ethical Consistency’ in Geoffrey Sayre-McCord (ed), *Essays on Moral Realism* (Cornell UP 1988) 41, 52.

70 ibid 49.

71 ibid 50.

72 Martha C Nussbaum, ‘Aristotle, Nature, and Ethics’ in JEJ Altham and Ross Harrison (eds), *World, Mind, and Ethics: Essays on the Ethical Philosophy of Bernard Williams* (CUP 1995) 86, 100.

73 Indeed, it only arises when ‘the time for supported decision-making is past’. *Re NRA* [2015] EWCOP 59 [56] (Charles J).

74 Series (n 5) 84.

75 *Aintree University Hospitals NHS Foundation Trust* v *James* [2013] UKSC 67 [18].

76 *Re MN* [2015] EWCA Civ 411 [80] (Munby P). This case has been granted permission to appeal to the Supreme Court.

77 Care Act (n 49) s 1(2)(d).

78 ibid s 2(1)(c).

79 ibid s 18(1).

80 The Care and Support (Eligibility Criteria) Regulations 2014, SI 2015/313, reg 2(2) The regulations do mention ‘accessing and engaging in work, training, education or volunteering’ —reg 2(2)(h)—but this refers to someone’s ability to access existing programs. There is nothing to indicate that it requires the provision of the support discussed here.

81 Care Act (n 49) s 19(1).

82 Isaiah Berlin, ‘Two Concept of Liberty’ in Henry Hardy and Roger Hausheer (eds), *The Proper Study of Mankind: An Anthology of Essays* (Pimlico 1998) 191, 203.

83 [2014] EWCOP 2, [2014] COPLR 439 [45] (Hayden J).

84 ibid [24], [59].

85 Select Committee (n 28) para 104.

